# Supplementary material for: C6 Ceramide Inhibits Canine Mammary Cancer Growth and Metastasis by Targeting EGR3 through JAK1/STAT3 Signaling
Source: Animals (Basel). 2024 Jan 27;14(3):422. doi: 10.3390/ani14030422 (PMC10854580; doi:10.3390/ani14030422)
Supplement: Supplementary file 1 [file animals-14-00422-s001.zip › animals-2763726-supplementary.pdf]

**Figure5A**

Ctrl C6-Cer Ctrl C6-Cer

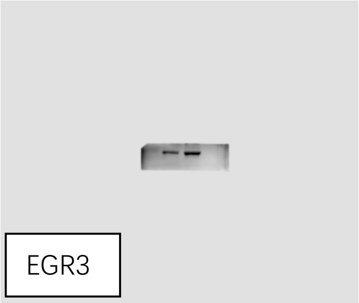


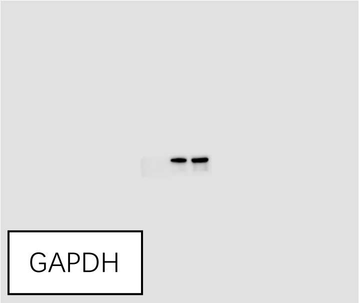


Marker Ctrl C6-Cer C6-Cer

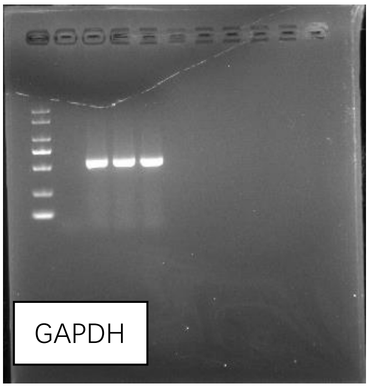


Marker Ctrl C6-Cer C6-Cer

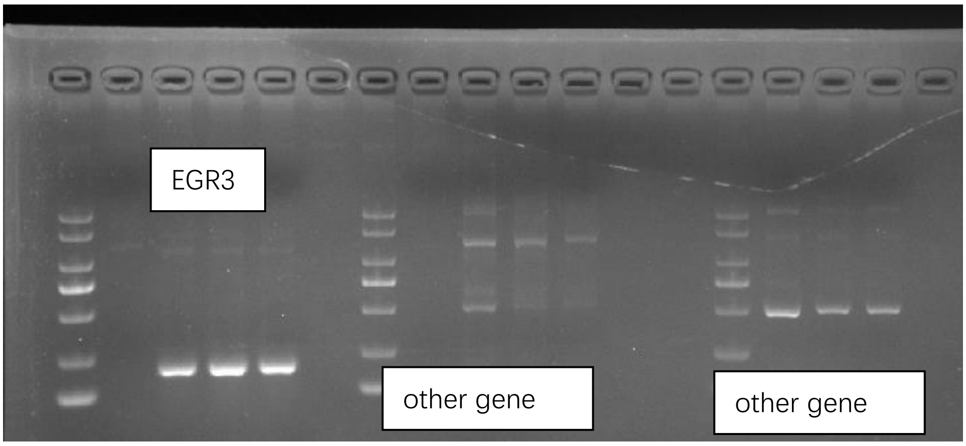

**Figure5D**

sh-luc sh-EGR3

Marker1 Marker2 sh-luc sh-EGR3

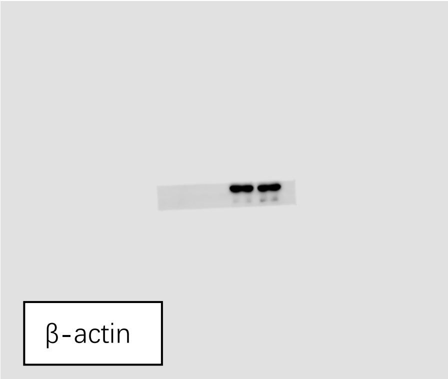


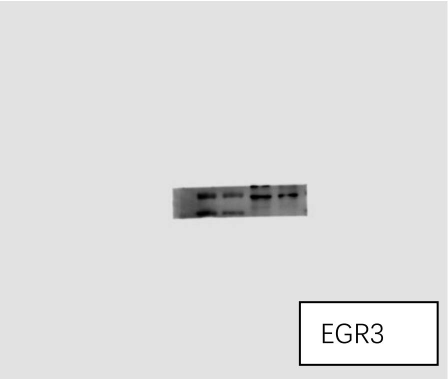


Marker sh-luc sh-EGR3 Marker sh-luc sh-EGR3

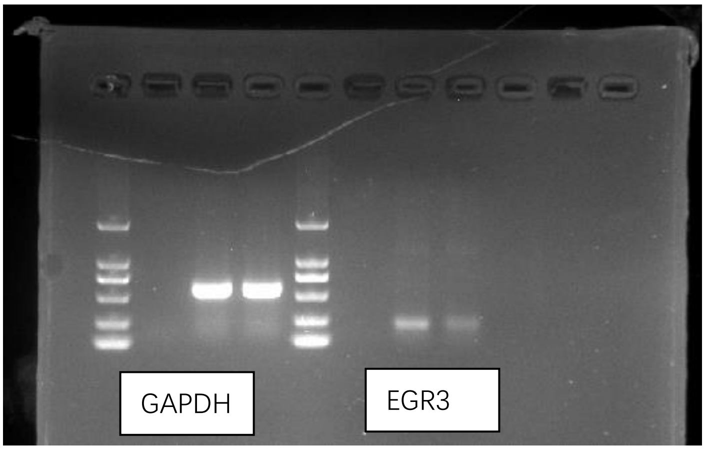


Figure 5K

sh-luc sh-EGR3

sh-luc sh-EGR3

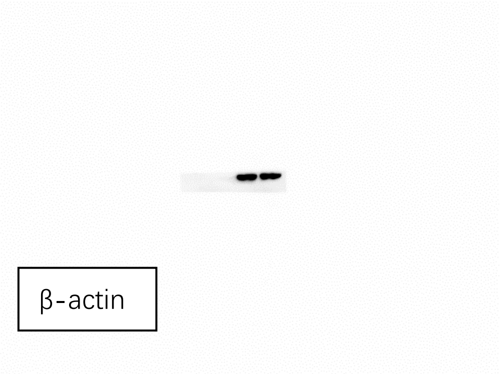


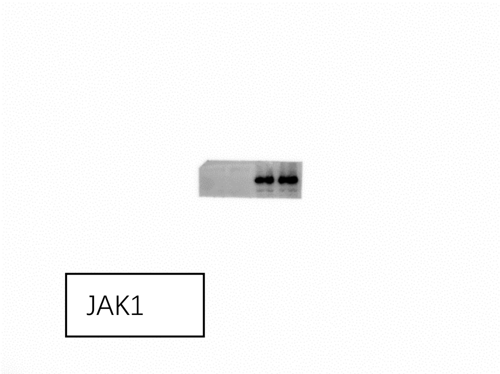

sh-luc sh-EGR3

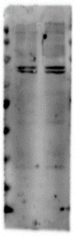

pJAK1

sh-luc sh-EGR3

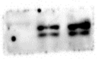

pSTAT3

sh-luc sh-EGR3

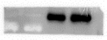

STAT3
